# Impacts of epidemic outbreaks on supply chains: mapping a research agenda amid the COVID-19 pandemic through a structured literature review

**DOI:** 10.1007/s10479-020-03685-7

**Published:** 2020-06-16

**Authors:** Maciel M. Queiroz, Dmitry Ivanov, Alexandre Dolgui, Samuel Fosso Wamba

**Affiliations:** 1 Postgraduate Program in Business Administration, Paulista University - UNIP, São Paulo, 04026-002 Brazil; 2 Supply Chain and Operations Management, Berlin School of Economics and Law, 10825 Berlin, Germany; 3 IMT Atlantique, LS2N - CNRS, La Chantrerie, 4 rue Alfred Kastler, 44307 Nantes, France; 4 Information, Operations and Management Sciences, TBS Business School, 1 Place Alphonse Jourdain, 31068 Toulouse, France

**Keywords:** Supply chain, COVID-19, Influenza, Resilience, Epidemic outbreaks, Pandemic, Structured literature review, Adaptation, Digitalization, Preparedness, Recovery, Ripple effect, Sustainability

## Abstract

The coronavirus (COVID-19) outbreak shows that pandemics and epidemics can seriously wreak havoc on supply chains (SC) around the globe. Humanitarian logistics literature has extensively studied epidemic impacts; however, there exists a research gap in understanding of pandemic impacts in commercial SCs. To progress in this direction, we present a systematic analysis of the impacts of epidemic outbreaks on SCs guided by a structured literature review that collated a unique set of publications. The literature review findings suggest that influenza was the most visible epidemic outbreak reported, and that optimization of resource allocation and distribution emerged as the most popular topic. The streamlining of the literature helps us to reveal several new research tensions and novel categorizations/classifications. Most centrally, we propose a framework for operations and supply chain management at the times of COVID-19 pandemic spanning six perspectives, i.e., adaptation, digitalization, preparedness, recovery, ripple effect, and sustainability. Utilizing the outcomes of our analysis, we tease out a series of open research questions that would not be observed otherwise. Our study also emphasizes the need and offers directions to advance the literature on the impacts of the epidemic outbreaks on SCs framing a research agenda for scholars and practitioners working on this emerging research stream.

## Introduction

The contemporary world has been challenged by unprecedented disease outbreaks (Chew et al. 2004; Lin et al. 2020; Nigmatulina and Larson 2009), whith significant negatively effects on the society as a whole, but also on the efficiency of operations and supply chain (SC) management (OSCM) business models. Such disruptive impacts frequently yield the ripple effects (Ivanov 2020a; Ivanov et al. 2018; Pavlov et al. 2019b). While SCs across the globe have been already suffering from epidemics and pandemic, they have recently been seriously hit by an unprecedented, far-reaching disruptive epidemic outbreak, namely COVID-19 (Boccaletti et al. 2020), which is considered as a new type of extremely contagious coronavirus, with destructive impacts (Choi 2020; Ivanov 2020a; Ivanov and Dolgui 2020b).

The COVID-19 was first reported in Wuhan, Hubei province, China, in the late 2019. As noted by the Johns Hopkins University on May 27, 2020, the number of confirmed cases reported around the world have been steadily growing, reaching 5.69 millions with 355,575 deaths (Johns Hopkins University & Medicine 2020). In view of this exponential growth, the COVID-19 was declared a world pandemic by the World Health Organization—WHO (2020) on March 11.

The impacts of the COVID-19 on SCs have already gained attention of scholars (Choi 2020; Govindan et al. 2020; International Journal of Production Research 2020a, 2020b; Journal of Operations Management 2020; Ivanov 2020a; Lin et al. 2020; Sarkis et al. 2020) and industry experts (Business Insider 2020; Deloitte 2020; Forbes 2020a, b; Fortune 2020; Harvard Business Review 2020; Institute for Supply Chain Management—ISM 2020). The COVID-19 epidemic is already impacting the OSCM at a large scale (Lin et al. 2020). Fortune (2020), in a report published on 21 February 2020, indicated that 94% of the companies listed in the Fortune 1000 list were facing SC disruptions due to the COVID-19. As for Deloitte (2020), the publication highlighted that the whole effect of the pandemic on SCs remained uncharted. The past epidemic outbreaks offer valuable lessons in relation to the SCs. The World Economic Forum—WEF (2020a, b) emphasized the need for firms and organizations to reengineer and adapt SCs to their future trade challenges. For instance, the short-term priority may be ‘transport and production’ and ‘worker movement’, while in the long term, capabilities and strategies related to ‘digital readiness & data sharing’ would be developed and implemented for SCs (World Economic Forum—WEF 2020a, b).

In a context where severe disruptions (e.g., manufacturers closed or partially closed, airports operating with harsh restrictions, shortages of medical equipment and supplies) are recorded in the global SCs (Ivanov 2020a; McKinsey & Company 2020; World Economic Forum—WEF 2020a), a good number of industries (automotive, electronics, medical equipment, consumer goods, etc.) also experience ripple effects (Dolgui et al. 2018; Ivanov 2020a, b). For example, as China is considered a world’s factory, the pandemic’s disruptions to SCs around the world started there before spreading elsewhere (Deloitte 2020). The severe ripple effects from this challenge requires different strategies and actions, including robust SC resilience strategies (Chen et al. 2019; Ivanov and Sokolov 2019; Pournader et al. 2020). Moreover, responses from the OSCM to such outbreaks by should consist in rendering global SCs more integrated and digitally ready (Choi et al. 2020; World Economic Forum—WEF 2020a, b). The digitalization of the SCs could improve the quality of the response to outbreak-related disruptions by enhancing the OSCM flexibility (Ivanov et al. 2019) in such circumstances.

It should be noted that the OSCM literature has approached studies on different types of epidemic outbreaks, with various objectives. For instance, outbreaks of influenza (Mamani et al. 2013), Ebola (Büyüktahtakın et al. 2018), Cholera (Anparasan and Lejeune 2017) and malaria (Parvin et al. 2018), among others, have been thoroughly studied. The majority of these papers have devoted particular attention to resource allocation, distribution of the medicaments, vaccines procurement policies, and emergency health response. It is true that recent advances have been made by the literature concerning SC response (Aldrighetti et al. 2019; Banomyong et al. 2019; Lu et al. 2017; Song et al. 2018; Shen and Li 2017) and epidemic outbreaks operations (Anparasan and Lejeune 2019; Chick et al. 2008; Long et al. 2018; Paul and Venkateswaran 2020), but the effects of epidemic outbreaks on SCs (Ivanov 2020a; Sarkis et al. 2020) are still to be adequately investigated. In this regard, operations research (OR) and operations management (OM) approaches such as network and complexity theories (e.g., Bayesian networks, Markov chains, network theory, ecological modeling) (Demirel et al. 2019; Hosseini and Ivanov 2019; Li and Zobel 2020), simulation (agent-based simulation, discrete-event simulation, systems dynamics) (Ghadge et al. 2013; Zhao et al. 2019; Ivanov 2020a), optimization (stochastic programming, robust optimization, mixed-integer linear programming, heuristics, dynamic programming) (Yoon et al. 2018; Amiri-Aref et al. 2019; Sawik 2019) could bring interesting insights to address this complex pandemic context. Moreover, empirical theories such resources based view (RBV), dynamic capabilities, contingency theory, organizational information processing theory (OIPT) with applications to resilience (Bode et al. 2011; Dubey et al. 2019b, c, 2020) could be employed jointly with OR/OM approaches to frame empirically-grounded analytics and to examine the impacts of epidemic outbreaks on SCs.

However, the literature reporting the application areas of these methods in a systematic way remains scarce. The results remain scattered across different sources and rather unsystemized. We could identify only one paper covering the literature review on the impacts of epidemic outbreaks on logistics (Dasaklis et al. 2012). Considering the paucity of the literature on the effects of epidemic outbreaks on SCs (Ivanov 2020a; Ivanov and Dolgui 2020b), our study intends to unlock and shed more light in this discussion, providing valuable insights that will enable decision-makers and policy-makers to develop their response plans for SCs. In contrast to Dasaklis et al. (2012), our study analyses the latest articles published by March 2020 and offers a broader picture by covering different areas of OSCM. Moreover, our ultimate objective goes beyond a pure literature review to identify what happended in the past, but we rather use the literature review results to project future research agenda for OSCM under the conditions of COVID-19 pandemic.

For the moment, this topic is still at a nascent stage, even though it is promising and crucial research area (Haren and Simchi-Levi 2020; Ivanov 2020a; World Economic Forum—WEF 2020a, b). Therefore, this work aims to help bridge the gap by investigating the available OSCM literature on the impact of epidemic outbreaks on SCs and logistics while proposing avenues for improvements. We seek to provide a robust research agenda that will contribute to advancing the extant relevant literature, feeding decision- and policy-makers with significant insights for the OSCM-related fields. Once again, the extant literature remains silent on the disruptive impacts of epidemic outbreaks on SCs, and this study is committed to help bridging this gap. More specifically, we are taking the lead of research by attempting to answer the following research questions (RQs).

### RQ1:

How does the OSCM literature address issues related to epidemic outbreaks in terms of their impact on logistics and SCs?

### RQ2:

What are the main problem settings and methods used?

### RQ3:

What are the open questions and future research opportunities framing OSCM at the times of COVID-19 pandemic?

The following contributions are expected from this study. The first and main contribution of our study is to map out an emergent research agenda for OSCM at the times of COVID-19 pandemic. The second contribution is a systematization of the literature investigating the interplay between epidemic outbreaks and SCs. The third contribution is an identification of several open research questions to be explored in very near future.

In terms of organization, Sect. 2 of this paper highlights the SLR methodology, supported by its findings and the content analysis in Sect. 3. Section 4 elaborates on a research agenda for OSCM at the times of COVID-19 pandemic. Section 5 serves as a conclusion and underscores other key considerations.

## Systematic literature review

Systematic literature review (SLR) has a long tradition, mainly in medical sciences (Cochrane Library 2018). The SLR is briefly defined as: “[…] an efficient technique for hypothesis testing, for summarising the results of existing studies, and for assessing consistency among previous studies; these tasks are clearly not unique to medicine.” (Petticrew 2001, pp. 99–100).

The SLR has been recently used successfully in the SC context (Mustafa Kamal and Irani 2014; Queiroz et al. 2019b; Roberta Pereira et al. 2014; Tachizawa and Wong 2014), which is eloquent evidence that, due to its systematic approach, the SLR is considered a rigorous method to conduct literature reviews. At the first stage of our approach, we therefore started with defining the scope and delimitating the subjects (Tranfield et al. 2003), taking into account their interdisciplinary nature. For the sake of objectivity and reliability in the whole process, we designed a research protocol (Table 1) (Tranfield et al. 2003).

**Table 1:** Research protocol

Research protocol	Details description
Research databases:	Scopus Database, ScienceDirect (Elsevier), Emeraldinsight (Emerald), Wiley Online Library (Wiley), Taylor & Francis Online (Taylor & Francis), Springer Link (Springer), Inderscience, and Informs PubsOnline
Publication type:	Peer-review journals (indexed by Scopus)
Language:	We considered only papers written in English
Date range:	The range period for consideration was 2003–2020 (March 22)
Search fields:	Titles, abstracts, and keywords
Search terms: applied in Titles in Scopus Database and in Titles, Abstracts, and Keywords in the other databases	(“outbreak*” OR “pandemic*” OR “epidemic*” OR “disease*” AND “humanitarian operati*” OR “humanitarian relief*” OR “suppl* Chain*” OR “logistic*”)
Criteria for inclusion	Papers that presented some outbreak in a logistics/SC context
Criteria for exclusion	Papers that presented outbreak discussion purely without protagonism of the logistics/SC, and review papers
Data extraction	We used an R-tool software Bibliometrix and the qualitative software MAXQDA
Data analysis and synthesis	Supported by the Bibliometrix and MAXQDA, we performed a content analysis approach

**Fig. 1:**
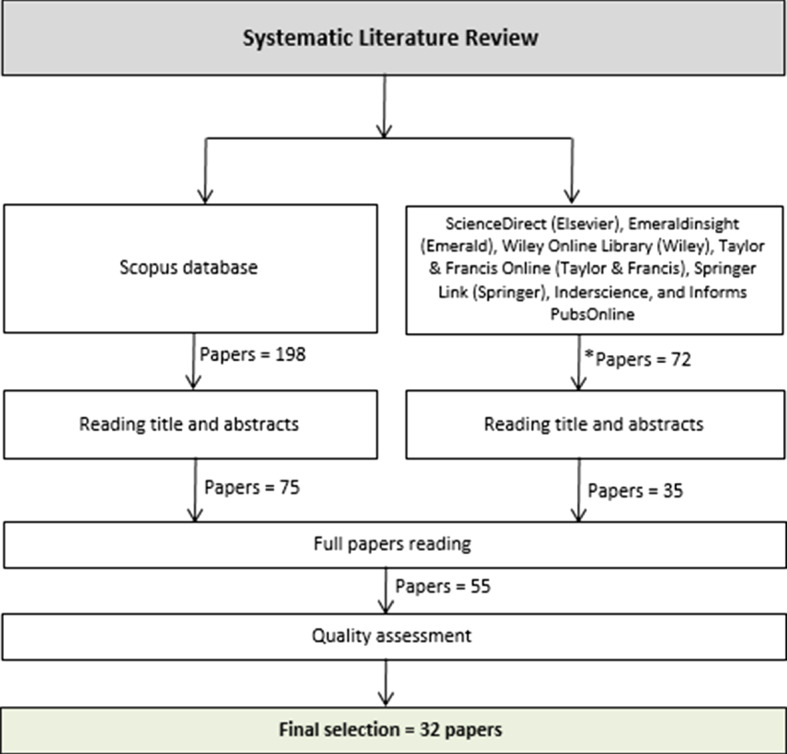
Research protocol to the SLR

At the second stage, we performed the search to identify the relevant papers. Following the recommendations (Tranfield et al. 2003), we considered only documents that met the full research protocol criteria. To comply with internal validity, we retained only journals indexed by Scopus. Moreover, to extract data and explore the papers, we used the quantitative software *MAXQDA* (Queiroz et al. 2019b; Schanes et al. 2018), and the *Bibliometrix*, an R-tool for science mapping and analysis (Aria and Cuccurullo 2017). At the third and last stage of the SLR, we reported the main findings from the analysis of papers. This led to unveiling several insights into both the knowledge literature and a number of research topics (Tranfield et al. 2003). Figure 1 highlights the main schematical flow of the review process.

### *Remark 1*

We note that our search was specifically designed in light of two aspects, i.e. (i) focus on commercial SCs and logistics (and not on humanitarian logistics) and (ii) focus on the evident and tangible logistics, production and SC contexts (i.e., it shoud be evident from the paper that it explicitly deals with an epidemic and SC issues; for example the papers that just mention epidemics as a possible risk were not considered).

### *Remark 2

In this query, due to the search considered title, abstract and keywords, we pre-selected only the first potential papers that met the Remark 1. Besides, forthcoming papers were also considered.

## Findings from the literature review

### Basic informations from the papers selected

As signalized in the previous section, we used an R-tool software application called Bibliometrix (Aria and Cuccurullo 2017; Zhou et al. 2019). The Bibliometrix is open-source application that has the potential to import data from different sources (Scopus, Clarivate Analytics’ Web of Science, among others). It has been used successfully in various literature review works (Bernardet et al. 2019; Demiroz and Haase 2019; Purba et al. 2019; Zhou et al. 2019), providing support for robustness and reliability in science mapping. Although we initially used the year 2003 in our searches, the first paper that met our research protocol dates back to 2008. However, with 12 years of time horizon, only 32 documents were selected considering all the inclusion criteria.

### Publications by journals

Table 2 shows the number of papers published in the course of each journal. The journals devoted to operations research (OR) topped the chart. Moreover, OR, operations management (OM) and logistics journals represent the majority of publications. They include publications such as Manufacturing and Service Operations Management, Journal of Humanitarian Logistics and Supply Chain Management, Production and Operations Management, Transportation Research Part E, among others. However, there are also influential medical/interdisciplinary journals such as the case of Lancet and CHEST.

**Table 2:** Articles published by the journal

Sources	Articles
European Journal of Operational Research	4
Annals of Operations Research	3
Journal of the Operational Research Society	2
Manufacturing and Service Operations Management	2
American Journal of Medicine	1
CHEST	1
Computers and Industrial Engineering	1
Computers and Operations Research	1
Influenza and Other Respiratory Viruses	1
International Journal of Integrated Supply Management	1
International Journal of Mathematics in Operational Research	1
International Journal of Production Research	1
International Journal of Systems Science: Operations and Logistics	1
Journal of Applied Poultry Research	1
Journal of Emergency Management	1
Journal of Humanitarian Logistics and Supply Chain Management	1
Management Science	1
Networks and Spatial Economics	1
Operations Research	1
PLOS Computational Biology	1
Production and Operations Management	1
Promet - Traffic - Traffico	1
Socio-Economic Planning Sciences	1
The Lancet	1
Transportation Research Part E	1

Moreover, our SLR identified the protagonism of the OR/OM journals while discovering that the number of papers on epidemic outbreaks was very limited. In the same light, journals dedicated to logistics and SCs were also in small number. Therefore, it becomes necessary to bridge this gap by producing more studies on the epidemic issues, including the COVID-19.

The top journals with regular papers published on these topics are European Journal of Operational Research (EJOR), Annals of Operations Research (ANOR), Journal of the Operational Research Society (JORS), and Manufacturing and Service Operations Management (M&SOM). These journals accounted for 34.38% of the publications. Concerning the different behaviors of these journals over time, it appears that the EJOR leads in terms of output, while the ANOR makes proof of a steady engagement, with one yearly publication over the last 3 years. It is noteworthy highlighting the recent interest for production research, SC and logistics by some dedicated journals, such as the International Journal of Production Research, the International Journal of Integrated Supply Management and Transportation Research Part E (with one paper in 2020* until March 18).

### Word TreeMap dynamics

**Fig. 2:**
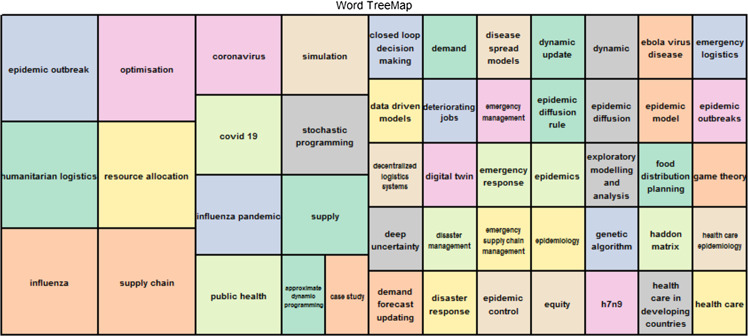
Word dynamics (keywords plus) per year

Figure 2 displays a Word TreeMap dynamics, based on keywords plus. It means that these words were not necessary given by the authors; instead, these were acquired from the titles of references from our dataset (32 papers). In addition, the frequency of the keywords determines the size of the rectangles, while the colors emphasize the relationship with the keyword. The role of word segments such as “epidemic outbreak,” “humanitarian logistics”, “influenza”, “optimization”, “resource allocation” and “supply chain” is quite identifiable. In the other rectangles (smaller sizes), we have various related words, but also emerging topics that are being already highlighted, such as “COVID 19”, “coronavirus”, “decentralized logistics systems”, “digital twin”, and “data-driven models.”

Moreover, there is a tendency for Coronavirus and COVID-19 to outperform each other over time, but we can also notice the growth of other interesting topics related to “Coronavirus and COVID-19”, such as “optimisation”, “epidemic outbreak”, humanitarian logistics” and “resource allocation”. In the other rectangles (smaller sizes) we have various related words.

Other related emerging topics can be unveiled using words and groups of words such as “COVID-19”, “coronavirus”, “decentralized logistics systems”, “digital twin”, and “data-driven models”. Taking into account the word dynamics, we can see that “emergency response” is a well-explored topic due to its variations (e.g., disaster response, disaster management, emergency management, emergency SC management, and emergency logistics). Therefore, with regard to our research questions and objectives, it clearly appears that related impact themes as indicated through the words ‘risks’ and ‘resilience’ (the most frequent words) are scarse. Therefore, our study advocates the use of these two words in order to see through the literature and investigate more deeply the impact of epidemic outbreaks on SCs.

### Multiple correspondence analysis

**Fig. 3:**
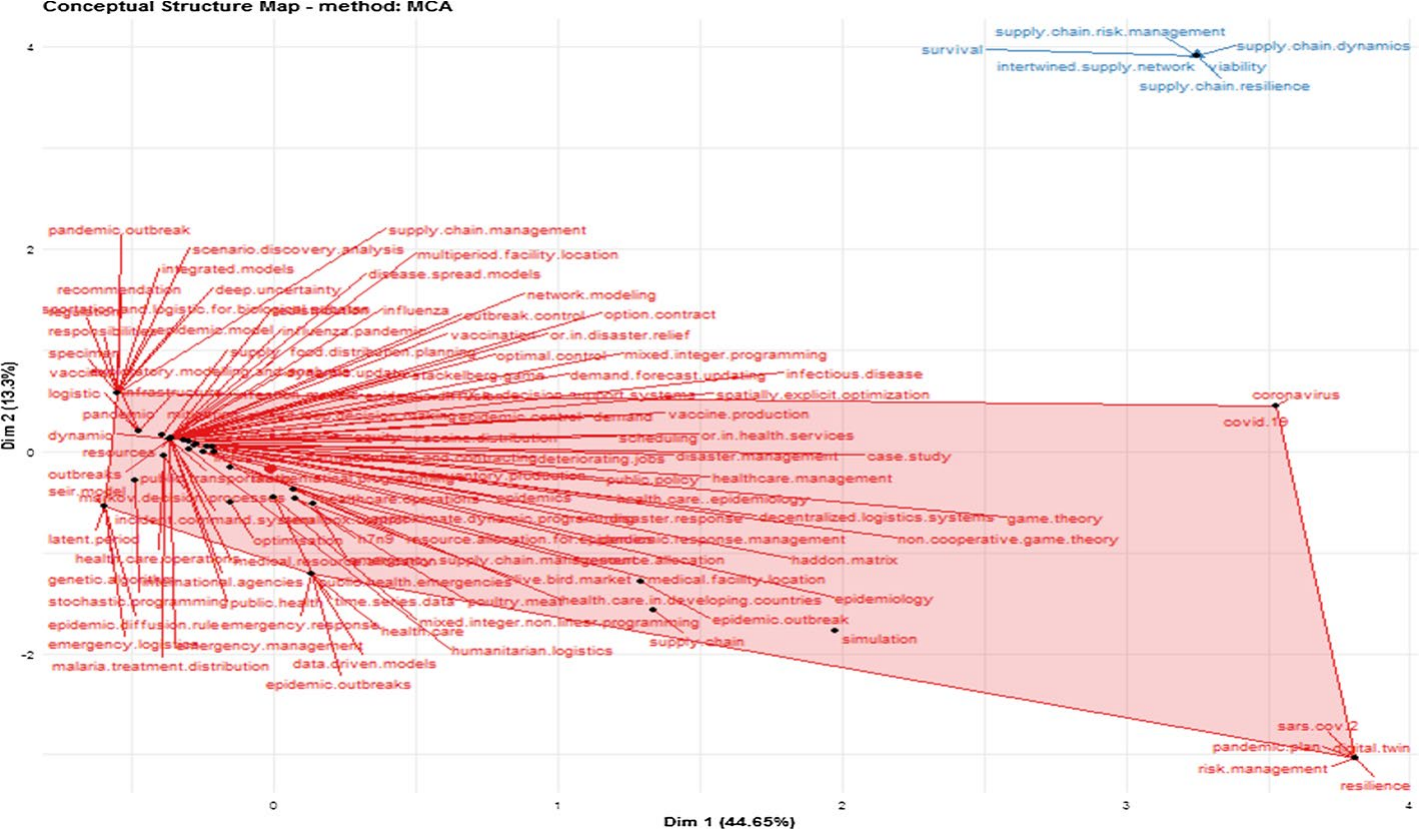
Conceptual structure map

In Fig. 3, we point out the conceptual structure map by employing a multiple correspondence analysis (MCA) method. The MCA is a data reduction technique that builds a map of the scientific conceptual structure (Aria and Cuccurullo 2017). Thus, in summary, the MCA plots a robust two-dimensional graphical map while taking into account the similarities of the distribution of the words used in its representation (Aria and Cuccurullo 2017; Cuccurullo et al. 2016). The more such words are similar and closer in the map, the better they will be represented.

In this regard, a concentration of optimization techniques is essential to provide an adequate response to epidemic outbreaks (e.g., optimization, Markov decision processes, stochastic programming, health care operations, mixed integer and non-linear programming, explicit optimization). Another interesting concentration is related to the susceptible exposed infectious removed (SEIR) model, epidemic diffusion rule, genetic algorithm, and latent period.

Also, the efforts related to epidemic response are represented by a significant concentration of “pandemic response management”, “emergency OSCM”, “public health emergencies”, “healthcare management”, “resource allocation”, and “vaccine distribution”, *inter alia*. Besides, there are other interesting concentrations related to “network modeling”, “transportation”, “demand forecast”, “data-driven models”, and “options contract”. Finally, blue words represent an emerging, challenging and unexplored topic (Ivanov 2020a, b) related to the COVID-19 pandemic. In this landscape, important words appear in this concentration, like “resilience”, “digital twin”, “risk management”, and “pandemic plan”.

### Categorization of epidemic and outbreaks in SCM

To synthesize our main findings from the literature review, we employed a content analysis technique (Kache and Seuring 2014; Queiroz et al. 2019b; Brandenburg and Rebs 2015) that could provide validity, objectivity and reliability through a coding-scheme approach, as recommended by previous studies (Queiroz et al. 2019b; Spens and Kovács 2006). The categories considered during the analysis of papers were Outbreak/Disease reported, Purpose, Main method/Theoretical approach, and Supply chain/Logistics/Operations implications. Table 3 highlights the findings from the content analysis.

**Table 3:** Content analysis categorization

References	Outbreak/disease reported	Purpose	Main method/Theoretical approach	Supply chain/Logistics/Operations implications
Rachaniotis et al. (2012)	Influenza	Proposing a deterministic scheduling model for resources allocation	Simple deterministic SIR model/Case study	Management of the scarce resources considering multiple member’s demands is too complicated. A deterministic model can support the strategies and policies to manage limited resources
Liu and Zhang (2016)	Influenza	Development of a logistics model to medical resources allocation considering different members of the SCs	Mixed-integer programming/FPEA model/Susceptible-exposed infectious-removed (SEIR)/Numerical example	The authors integrated a time-series demand approach model with logistics planning considering orders, shipping, and resource allocation. Thus, it found a significant minimization of the forecast error, reflecting improvements in the SCs
Mamani et al. (2013)	Influenza	Analysis of the vaccine allocation inefficiency and contractual mechanism model proposing (vaccine procurement decisions) in SCs	Hybrid epidemic model/Game theory/Numerical experiments	Imbalance in the integrated and coordinated global SCs vaccines impacts on the shortfall or excess, and thus on the SC costs. Therefore, the utilization of the coordinated contract could generate benefits (e.g., costs and shortages reduction)
Büyüktahtakın et al. (2018)	Ebola	Proposing a logistics epidemic model to controlling Ebola epidemic, considering the location of the resource	Mixed-integer programming (MIP) model/Case study/Secondary data (WHO)	Introduction and validation of an optimized epidemics logistics model to resource allocation in the SCs, considering the constraints of the treatment centers. In addition, the model provides useful information while taking into consideration the geographical parameters, the dynamics of the infected in different regions and the impact on resources allocation
Majić et al. (2009)	Influenza	Analysis of the airport infrastructure and logistics procedures for the distribution of medicaments	Secondary data analysis (WHO)	The logistics infrastructure plays a fundamental role to refrain the epidemic, but the infrastructure has huge challenges. Besides, rigorous quality control is needed to handle the medicaments safely
Shamsi et al. (2018)	Epidemic/outbreak control	Development of a model based on an option contract to enhance supply vaccines (procurement) and minimize the social costs	SIR epidemic model/Stackelberg game model/Non-linear programming/Numerical experiments	The option contract model can help the suppliers to establish the optimal values of the vaccines demanded, as well as the buyer’s forecasting, thus leading to the minimization of procurement and social costs
Anparasan and Lejeune (2017)	Cholera	Proposing a novel Haddon Matrix to support the response to the epidemics	Literature review/Haddon Matrix	A framework that provides useful insights into humanitarian logistics operations at all stages (pre-event/response/post-event
Bogoch et al. (2015)	Ebola	Analysis of the Ebola diffusion by international air travelers and the airport’s infrastructure role to combat it	Secondary data analysis (WHO, World Bank, US Centers for Disease Control and Prevention)	Executing the passengers screening in the airport of origin seems an efficient method; however, the logistics infrastructure can be a limitation
Anparasan and Lejeune (2018)	Cholera	Development of a data-driven model based on data set to support epidemic control policies and emergency health response	Secondary data analysis (WHO, Centers for Disease Control and Prevention (CDC), and Ministry of Health and Population of Haiti (MSPP))/Integer linear programming	The data-driven analysis enables robust SC models for resource allocations and emergency response, while empowering the medical staff in an integrated and coordinated chain
Muggy and Heier Stamm (2020)	Cholera	Proposing two models for humanitarian SCs response, considering the distance and congestion to individual decisions related to facility locations to supplies/services	Beneficiary decision model/Centralized planner’s model/Network congestion games/Player-facility-specific congestion weights problem (PFSCWP)/Secondary data	The authors showed that the proposed models could support post-disaster response (cholera control in this case) by supporting the public health SC’s, especially in the resource allocation. The models showed the importance to consider the effects of the individuals’ behavior in humanitarian SCs to pursue the optimality of the network
Parvin et al. (2018)	Malaria	Development of a methodology for the distribution of drugs while considering the strategic and tactical aspects of a three-tiers health system	Stochastic programming/Cluster/Markov decision/Case study	The models showed that efficient transportation planning can contribute to significantly reducing costs and shortages. However, implementation is severely impeded by challenges like the lack of communication, weak government efforts and engagement, and poor logistics infrastructure
Savachkin and Uribe (2012)	Influenza	Proposing a drug distribution model by considering dynamic strategies (redistribution of the resources, considering the pandemic behavior)	Simulation/Optimization model	The proposed model can redistribute medicaments in a dynamic way, taking into account the progress of the outbreak. The model also considers logistics factors (costs, distance, resources availability etc.) and the progression of the pandemic
Dasaklis et al. (2017)	Smallpox	Proposing a model for responding to smallpox through SC emergency, considering a large-scale vaccination scenario	Linear programming/Numerical experiment	The configuration and operationalization of howemergency network response impacts directly on the outbreak control, and consequently, in the entire activities (social and economics). A model was developed, taking into consideration two stages (i. pandemic progress; ii. the distribution model). The authors point out the influence of the resources available to control the epidemics
Bóta et al. (2017)	Epidemic/outbreak control	Proposing a network structure to vehicle trip considering the patterns of the number of passengers	Simulation/Secondary data/Case study/SIR model	Identification of the infectious vehicle trip network could support logistics and SC strategies for planning the distribution and avoiding risky trips. The authors proposed a model that promotes the identification of a public system that is the most propensity to transport passengers contaminated
Liu et al. (2019)	Influenza	Proposing an epidemic logistics model for controlling H1N1, based on the Büyüktahtakın et al. (2018) model	Mixed-integer non-linear programming model (MINLP)/Case study	The proposed model found similarities and contrasts with the model proposed by Büyüktahtakın et al. (2018). The model support decisions about when the isolated and unused wards may be open or closed
Tao et al. (2018)	Epidemic/outbreak control	Development of a vaccination distribution model, considering an intermediate optimal solution	Stochastic-SIR model/Simulation	The logistics resources available can determine an intermediate solution as the “best”, in view of the high constraints of the logistics resources
Ekici et al. (2014)	Influenza	Modeling food distribution planning to combat the influenza pandemic	Agent-based continuous-time stochastic model/Mixed integer linear programming/Heuristics development	The model showed proof of robustness to support food demand planning in the network, facility location and resource allocation/distribution. Also, the authors found that voluntary quarantine can help several industries to cope with capacity issues. For instance, food distribution facilities and SCs could operate by almost half of their capacity
Sun et al. (2014)	Influenza	Development of optimization models for patient and resources allocation	Optimization models/Case study	The proposed models can help the logistics and SC health-care systems to plan and manage resources efficiently. Besides, the models can help decision-makers to avoid resources shortage
Enayati and Özaltın (2020)	Influenza	Proposing a vaccine distribution model considering the minimization of the vaccines necessary to contain the pandemic and equity criteria to coverage subgroups	Mathematical programming model/Exact discretization with multiparametric disaggregation method	The proposed model can support public health decision-makers concerning vaccine storage logistics to control outbreaks. The policies to vaccine allocation reflect directly in the resource allocation efficiency. Moreover, policies for an equitable vaccination coverage can positively influence outbreak elimination
Cruz et al. (2015)	Influenza	Reporting the logistics challenges in the public health response, while considering international cooperation and support to response to the outbreak	Case study	The logistics activities play an essential role in providing an effective intervention in epidemic/outbreaks control. While logistics can fundamentally contribute to response quality, international logistics cooperation plays an essential role in the response process
Anparasan and Lejeune (2019)	Cholera	Proposing a model for epidemic response, considering the resources constraints (size, number, location of the facilities, staff, transportation, etc.)	Secondary data (Ministry of Health and Population of Haiti, WHO, Centers for Disease Control and Prevention)/Algorithm/Integer linear programming/Framework	The proposed model is capable of capturing the particularities of the country’s limited supply resources and to better help decision-makers to configure resource allocation to respond effectively to an epidemic. The model can also establish several types of resources (number of facility location, staff, transportation, patients to treatment, etc.)
Long et al. (2018)	Ebola	Development of an optimized model that operates at two levels: i) assign treatment units in regions; ii) compare four strategies to programming the units the affected locals	Heuristic/Myopic linear programming/Estimation–optimization/Approximate dynamic programming algorithm	Resource optimization contributes directly to epidemic control and thus to saving more lives. The authors highlight a resource allocation strategy that relies on anticipating future cases, known as forward-looking. Based on data, this strategy can render resource allocation dynamically attractive
Chick et al. (2008)	Influenza	Proposing an optimized model for SC vaccines dynamics, considering the coordination between contractual actors	Game theory/Optimization	The lack of coordination between the actors (government and manufacturers) can undermine the entire system and bring about vaccines shortfalls. According to the authors, while the global social optimum is hard to achieve, a contract based on cost-sharing between the parties (government and manufacturers) can impulse an optimum social accomplishment
Einav et al. (2014)	Epidemic/outbreak control	Development of insights to support efficient response to pandemic and disasters	Panel	The OSCM capabilities are critical for an effective care response. Resource distribution strategies are fundamental for an adequate response due as the demand seems higher than the available capacity. Besides, distance may appear as a barrier to logistics
Orenstein and Schaffner (2008)	Influenza	Presentation of the lessons learned about the Influenza logistics and SC management, considering the vaccines production, distribution, and management	Conceptual	The logistics and SCs are decisive in supporting public health humanization. However, these activities need full ownership by policymakers to avoid shortages
Wang et al. (2009)	Epidemic/outbreak control	Development of an emergency model for supply networks that takes into account the latent period influence in the demand during the epidemic	Multi-objective stochastic programming model/SEIR epidemic diffusion model/Optimization/Genetic algorithm/Monte Carlo simulation/Numerical example	To develop optimal solutions for the emergency medicine distribution, latent periods exert a huge constraint, causing delays and amplifying the uncertainty. One strategy to be considered is the allocation of the materials by employing collaboration between areas, in an integrated way
Hessel (2009)	Influenza	Discussion for vaccines SC planning, production and distribution, with related challenges	Conceptual	The fight against the pandemic requires a high-level involvement of policymakers in the entire SCs to plan and develop the required logistics capabilities at all stages. Thus, all members of the OSCM should operate in an integrated model with the governments
Paul and Venkateswaran (2020)	Epidemic/outbreak control	Proposing policies for mitigating the effects of the epidemic while considering deep uncertainty	Exploratory modelling and analysis (EMA) methodology/Machine learning/Scenario discovery/Supply shortage model	The SC plays a fundamental role in the epidemic’s control by ensuring an adequate flow of medicaments and minimizing shortages. In this regard, the period covered by the epidemic can be influenced by the SC activities, especially in relation to resources shortages
Khokhar et al. (2015)	Influenza	Analysis of the SC distribution of the chicken meat and its influence on the spreading of H7N9	Secondary data (China Animal Industry Yearbook/China’s Center for Disease Control and Prevention/China National Health and Family Planning Commission)	The suppliers and retailers need to work in a more integrated model. Cutting-edge technology systems can cope with this challenge. For instance, all SC members (including governments) can put in more traceability measures
Ivanov (2020a)	Coronavirus (COVID-19)	Prediction of the impacts of epidemic outbreak on SCs	Case study/Simulation	The epidemic outbreaks exert a destructive effect on SCs. The development of strategies to predict such impacts in different time horizons can support the performance of the SC and mitigate any adverse effects. A simulation is a powerful approach as it enables us to compare wrong and successful elements in the SC response plan
Ivanov and Dolgui (2020b)	Coronavirus (COVID-19)	Discussion and analysis of the intertwined supply network, considering survivability and resilience in the COVID-19 context	Conceptual/Game-theoretical model	The COVID-19 outbreak is forcing supply networks operate with different and robust resilience approaches. The paper indicates that intertwined supply networks (highly interconnected and resilient networks) need to be viable to guarantee long-term survivability effects, especially in exceptional events
Ivanov and Das (2020)	Coronavirus (COVID-19)	Investigation of the SC resilience in a COVID-19 disruption scenario	Simulation	The full range of COVID-19 disruptions in SCs remains unknown. In this paper, the auhtors provide valuable and unique insights to better mitigate risks related to the COVID-19 and reinforce resilience to the pandemic. They further highlight the value of creating flexible, redundant and real-time SCs in order to dynamically realocate demand and supply

### Discussion of findings

The categorization of the selected papers by the content analysis unlocked exciting findings. Firstly, we identified that the majority of papers were devoted to the Influenza epidemic outbreak (43.75%), followed by papers without any particular focus on a specific epidemic/outbreak, but with some insights into epidemic/outbreak control (18.75%). Then, Cholera and Ebola held the third and fourth rank (12.5%), followed by COVID-19 (9.38%), Malaria, and Smallpox, with one paper dedicated to these epidemics.

Furthermore, the selected papers mostly presented optimization models for resource allocations (medicaments and vaccines distribution, vaccines procurement contracts, patient allocation, facilities location, etc.). However, some studies approached these epidemic outbreaks for other purposes. This is the case of Bogoch et al. (2015), which considered the impact of Ebola diffusion on international air travelers and on the logistics infrastructure network that is required to minimize the spread. It is also the case of a paper by Ivanov (2020a, b) that addressed the COVID-19 impacts on SCs and how to predict it by employing simulation techniques (Ivanov 2020a; Ivanov and Das 2020; Ivanov and Dolgui 2020b).

Regarding the “Main Method/Theoretical Approach” reported by the papers, it should be recalled that most papers were related to optimization models, and therefore, they used different mathematical models and approaches. They included mixed integer programming (Liu and Zhang 2016; Büyüktahtakın et al. 2018), linear programming (Dasaklis et al. 2017), game theory (Shamsi et al. 2018; Chick et al. 2008), case study and simulation (Ivanov 2020a, b), case study and SIR model (Rachaniotis et al. 2012), case study and stochastic programming/Markov (Parvin et al. 2018), among others. Table 4 shows the distribution of papers by type of epidemic outbreak.

**Table 4:** Analysis of papers by a content analysis approach

Epidemic/outbreak reported	Number of papers	Percentage
Influenza	14	43.75
Epidemic/outbreak control	6	18.75
Cholera	4	12.50
Ebola	3	9.38
Coronavirus (COVID-19)	3	9.38
Malaria	1	3.13
Smallpox	1	3.13
Total	32	100.00

Lastly, concerning the “Supply chain/Logistics/Operations implications”, several interesting points were raised. For example, resource management is a great preoccupation (Rachaniotis et al. 2012; Savachkin and Uribe 2012; Long et al. 2018). In this regard, it should be noted that logistics and SCs play an essential role in coordinating and integrating the multiple members’ activities (Mamani et al. 2013), including manufacturers, transportation, hospitals, government, etc. Moreover, in a recent study, Paul and Venkateswaran (2020) emphasized the impact of the SC in order to control epidemic outbreaks. The authors pointed out that the SCs should provide an adequate flow of medicaments and other products to avoid materials shortage. Thus, SCs can influence the duration of an epidemic outbreak.

Recent studies by Govindan et al. (2020) and Ivanov (2020a) also presented the devasting impact of the COVID-19/SARS-CoV-2 on global SCs. This does not mean that interesting and new findings on the impact of COVID-19 on SCs have not been obtained. For instance, SCM decision-makers are henceforth well equipped to make use of the available technologies and relevant techniques (including simulation) to predict impacts on their organizations/firms’ SCs. Besides, analytics techniques to support decision-making in logistics operations (Griffith et al. 2019; Dubey et al. 2019b), and social media analytics to support emergency operations decisions (Fosso Wamba et al. 2019), play an essential role in order to minimize the epidemic impacts on SCs. Moreover, it is well demonstrated that the SC performance response relies on the scale and timing of the disruption spreading and not on the upstream disruption duration. Ivanov (2020a) went on identifying a positive effect of disruptions on SCs responses (upstream and downstream sides) to an epidemic, when the disruption is simultaneous.

## Research agenda for operations and supply chain management at the times of COVID-19 pandemic

This section deals with the main theoretical and managerial implications of our study with the objective to tease out a research agenda for OSCM at the times of COVID-19 pandemic. To start, Table 5 synthesizes open research questions following the identified literature gaps and makes important suggestions to scholars and practitioners.

**Table 5:** Proposed research agenda for investigating the effects of epidemic outbreaks on SCs

Literature gap	Open research questions (ORQ) and opportunities	Related literature	Cluster	Examples of OR/OM/miscellaneous approaches to support the ORQ
Models for sustainable operations in vulnerable SCs due to the epidemic outbreak. Development of sustainable SCs and production systems	How can sustainable operations models assist vulnerable SCs, especially in the developing economies, to minimize the supply effects (e.g., shortages, abusive prices)?	Sarkis (2012), Sarkis et al. (2020) and de Camargo et al. (2019)	Modeling	Mixed-integer linear programming Game theory Dynamic capabilities
Circular economy (CE) to mitigate the insufficient supply and production capacities	How could CE contribute to minimizing the effects of the production and supply shortages in global SCs?	Chiappetta Jabbour et al. (2020) and Sarkis et al. (2020)	Modeling	Complexity theory Systems dynamics
New optimization models for resource allocations in dynamically changing environments with consideration of smart cities	How can smart cities promote new resource allocation models to support the dynamic allocation in epidemic epicenters?	Israilidis et al. (2019) and Qi and Shen (2018)	Modeling	Robust optimization Stochastic programming
Optimized response of humanitarian operations	How can the global SCs prepare and maintain a flexible humanitarian operation response plan to a pandemic crisis anywhere in the world?	Baidya and Bera (2019), Çankaya et al. (2019), Fosso Wamba (2020), Rodríguez-Espíndola et al. (2018) and Turrini et al. (2019)	Modeling	Systems dynamics Mixed-integer linear programming Sociotechnical systems
Home care drones to minimize the transportation lead-time in essential medical supplies	How can drones be used to provide a prompt response in the supply network (distribution centers, hospitals, home) so as to transport medical samples and medicaments to quarantined people?	Pulver and Wei (2018) and The Ebola Gbalo Research Group (2019)	Modeling	Bayesian networks Markov chains Petri nets Dynamic capabilities
New and severe disruption effects on SCs	What are the worst and most severe disruption effects on SCs that lead to a ripple effect amplification?	Ivanov (2019a, 2019b, 2020a) and Pavlov et al. (2019b)	Modeling/organizational	Agent-based simulation Discrete-event simulation Organizational information processing theory
Co-benefits generated from the harmful effects of the epidemic outbreaks in SCs	What co-benefits can be brought from negatively impacted SCs within the society and within organizations? For instance, water pollution minimization, CO2 emissions, etc.	Cao et al. (2019) and Giordano et al. (2020)	Organizational	Agent-based simulation Discrete-event simulation Knowledge-based systems
Cannibalization of global SCs (e.g., countries fighting for medical supplies)	How does the cannibalization of global SCs create an impact in the short and long-term perspectives? What is the role of resilience in minimizing the impact of cannibalization?	Euronews (2020), NZ Herald (2020), The Wall Street Journal (2020) and Vatican News (2020)	Organizational	Systems dynamics Discrete-event simulation Contingency theory
Relocalization of manufacturing firms and the impact on outsourcing strategies	What are the effects of relocating manufacturing firms (e.g., coming back to or leaving China)?	Forbes (2020a) and United Nations Conference on Trade and Development—UNCTAD (2020)	Organizational	Discrete-event simulation Systems dynamics Dynamic programming Resource-based view
Investigation of the blockchain’s contribution in the minimization of the impacts of epidemic outbreaks on SCs	How can blockchain technologies support a responsive traceability system to avoid or mitigate the effects of shortages on SCs?	Dubey et al. (2020) and Wamba and Queiroz (2020)	Technology	Complexity theory Reliability theory Dynamic capabilities
Review of artificial intelligence (AI) techniques to support SC models in epidemic contexts	How can AI techniques contribute to developing responsive SC models in epidemics scenarios?	Fragapane et al. (2020), Bag et al. (2020) and Dwivedi et al. (2019)	Technology	Systems dynamics Discrete-event simulation Organizational resilience
3D printing to replace the missing suppliers	What are the main applications of 3D printing to replace urgent missing supplies during an epidemic outbreak? e.g., Personal Protective Equipment (PPE), mechanical ventilators to combat COVID-19, among others	Beltagui et al. (2020) and Tatham et al. (2015)	Technology	Complexity theory Systems dynamics Diffusion of innovation theory

We organized the literature gaps and open questions in three clusters, namely: the modeling cluster, the organizational cluster, and the technology cluster. The modeling cluster covers optimization, simulation or other OR methods. In the organizational cluster, we consider different forms of SC organisation, including intertwined supply networks (Ivanov and Dolgui 2020b), to improve collaboration with SCs and their interrelation with the society, etc. For example, the co-benefits generated by epidemic outbreaks are practically unknown, along with the cannibalization of global SCs and the relocalization of manufacturing firms. The technology cluster is dedicated to digital technologies, additive manufacturing, and data analytics. For instance, it addresses the question of how to use disruptive technologies to manage SC disruptions during epidemic crises.

Besides, in the last column of Table 5, we highlighted some suggested and adherent theories of operations (OR/OM) and miscellaneous theories to support the examination of the challenging open research questions (ORQ). In this vein, we identified from our literature analysis suitable OR/OM approaches such as network and complexity theories (e.g., Bayesian networks, complexity theory, reliability theory, petri nets, and Markov chains), mathematical optimization (e.g., mixed-integer linear programming, stochastic programming, metaheuristics, robust optimization, and dynamic programming), and simulation (e.g., agent-based simulation, discrete-event simulation, and systems dynamics). Moreover, these approaches could be integrated with empirical theories in exploring operations such as dynamic capabilities, absorptive capacity, organizational resilience, sociotechnical systems, organizational information processing theory, knowledge-based systems, contingency theory, resource-based view, and diffusion of innovation theory.

In that context, these theories could bring valuable support in exploring the impact of epidemic outbreaks on SCs. For instance, the mathematical optimization theories can be used develop optimized plan and schedules to avoid shortages, and also deal with ramp-up demands (e.g., toilet paper manufacturers ramping up production due to COVID-19). Simulation techniques allow to develop robust resilience plans, considering new and severe disruptions (e.g., agent-based simulation allows to examines operations behaviours taking into account simultaneous interactions of multiple agents, during and after the COVID-19). Furthermore, due to the complexity of the COVID-19, these approaches could be integrated with some network and complexity theories (e.g., combining some simulation theory with reliability analysis, decision-makers could develop complex scenarios to cope with resource scarcity and simultaneous supply and demand substitutions required at each stage of a pandemic).

**Fig. 4:**
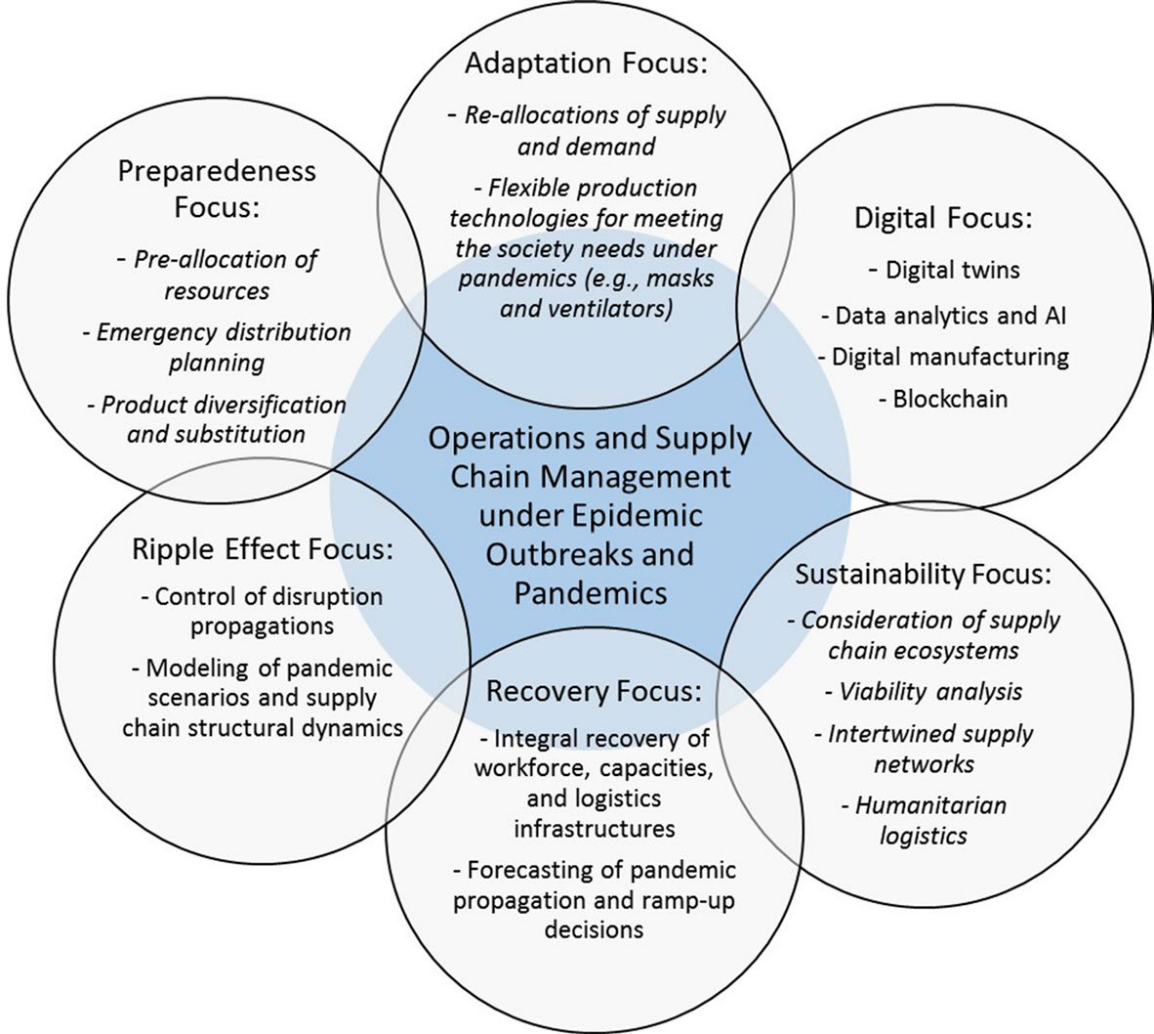
Emerging research agenda on OSCM under pandemics and epidemic outbreaks

In Fig. 4, we summarise the results of our analyses as an emerging research agenda on OSCM under pandemics and epidemic outbreaks.

The research agenda in Fig. 4 is teased out the Tables 3 and 5. It shows open research questions as an extension of our findings during a systematic literature review. Now we discuss the individual components presented in Table 5 and Fig. 4 in detail.

### Preparedeness focus

Pre-allocation of resources and emergency distribution planning have been identified in our our SLR as the most visible topics with the preparedeness focus whereas optimization methods dominate this area. In this sense, the research opportunities relate not only to optimization models, but also to the need to implement more sophisticated simulation techniques (Currie et al. 2020). In addition, queuing theory, scheduling and forecasting approaches need to be explored in pandemic contexts. Our study sheds more light on the importance of managing and allocating resources in an adequate way (Pavlov et al. 2019a). This means that decision-makers and policy-makers should improve their organizations’ SC resilience and response capacity by employing not only optimization techniques but also simulations (Paul et al. 2019). First, an in-depth understanding of status quo in SC responsiveness and resilience can be achieved through simulations (Currie et al. 2020; Ivanov 2020a; Ivanov and Das 2020). Second, simulation is essential to predict massive disruption scenarios and the required performance capacities.

### Digital focus

Questions related to cutting-edge technologies like blockchain and AI techniques, for improved response traceability, are fundamental to provide robust, resilient SC models. On the other hand, there is an urgent need to explore the 3D printing/additive manufacturing to efficient and timely deployment of medical equipment. Moreover, the first analyses of COVID-19 pandemic impacts on SCs and production systems suggest that Industry 4.0 and digital manufacturing can play a critical role for SC resilience and ripple effect control (Ivanov and Dolgui 2019; Hosseini et al. 2019). In terms of visibility and digital control, the firms that are successful in digital manufacturing networks seem to be better positioned in crisis times and in the coordination of future recovery processes (Choi et al. 2020; Dubey et al. 2019a; Ivanov et al. 2019; Panetto et al. 2019; Ivanov 2020a, b; Ivanov and Dolgui 2020a, b; Ivanov and Das 2020; Ni 2020).

### Adaptation and recovery focus

We found several tensions related to SC responses to past disruptions caused by epidemic outbreaks. From the theoretical perspective, we found that the OSCM literature has addressed the past disruptions caused by the epidemic outbreaks by employing mainly optimization approaches, especially in relation to resources allocation (Büyüktahtakın et al. 2018; Liu and Zhang 2016; Parvin et al. 2018; Rachaniotis et al. 2012). Managers and practitioners need to continuously monitor SCs, as no stage of epidemics’ impacts should be left unturned before any recovery plan is properly implemented and managed. To this end, one of the main lessons from the extant literature is that the OSCM field exerts a great influence on the duration of epidemics (Paul and Venkateswaran 2020). Therefore, the OSCM should operate from the resilience perspective (Dubey et al. 2019c; Dolgui et al. 2020), avoiding medicament and equipment shortages, providing a dynamic and responsive operation model at the different stages of the outbreak. In addition, recent studies on the disruptions of SCs provoked by epidemic outbreaks showed that simulation is an important technique to predict and develop plans to respond to such impacts on OSCM (Currie et al. 2020; Ivanov 2020a; Ivanov and Das 2020; Ivanov and Dolgui 2020b).

### Ripple effect focus

Our next finding is that epidemic outbreaks could significantly impact the SCs triggering a highly and unexplored ripple effect (Ivanov 2020a). The main important theoretical implication resides in reinforcing the need to deepen the understanding of *the simultaneous deployment of the ripple effects and epidemic outbreaks on SCs* (Ivanov and Das 2020; Ivanov and Dolgui 2020b). The ripple effect is a very strong stressor to SCs and their ongoing collapses amid pandemic coming from the disruption propagations through the networks. Adversely, the situation is stimulated by simultaneous disruptions and uncertainties in demand and supply. The existing knowledge in modelling the SC ripple effect (Dolgui et al. 2018) is multi-faceted and deserves to be analysed for the unique set of factors shaping SC adaptations during and after a global pandemic.

### Sustainability focus

The sustainability focus builds around consideration of SC ecosystems and viability. An SC can be considered viable if it is able to maintain itself and an ecosystem balance (i.e., achieve homeostasis) (Ivanov and Dolgui 2020b). Sarkis et al. (2020) point to the potentials of the circular economy to ensure long-term SC survivability. Moreover, there are multiple feedback cycles in the SC ecosystems, including both positive and negative feedbacks. As pointed in Ivanov (2020b), the interactions of the SC and nature are concerned with a positive cycle of using natural resources and a negative cycle of emissions as potential contributors to climate change. The interaction with society results in positive feedbacks such as technological innovations and workforce development although negative feedbacks in terms of possible labor strikes (disruptions at SC resilience level) or global pandemics (disruptions at SC survivability level) also exist. In this vein, the analysis can be brought to the levels of intertwined supply networks (ISN), i.e., “the entirety of interconnected supply chains which, in their integrity secure the provision of society and markets with goods and services” (Ivanov and Dolgui 2020b). Finally, the issues of humanitarian logistics and SCs build a central perspective in the sustainability focus (Besiou and van Wassenhove 2020; Dubey et al. 2020; Fosso Wamba 2020). From this perspective, additional research is essential to address concerns of process improvement in humanitarian operations (Larson and Foropon 2018).

### A discussion note on SC resilience at the pandemic times

Based on our findings, we propose a classification of relevant aspects of SC resilience, in view of the particularities of some epidemic outbreaks and global pandemics. The results of our analysis show that the traditional SC risk and resilience understanding (Tang 2006; Govindan et al. 2017; Chen et al. 2019; Dolgui et al. 2018; Ivanov et al. 2018; DuHadway et al. 2019) is restrictive in tackling long-term, global pandemic disruptions. Therefore, we call for new approaches (Ivanov 2020a; Ivanov and Das 2020; Ivanov and Dolgui 2020b) or for the extension of the existing ones. Based on the categorization proposed by Ivanov and Dolgui (2019), we therefore propose to categorize the SC resilience actions in the context of pandemic disruptions, using four main categories, namely: systems, process, control, and recovery, as reported in Table 6.

**Table 6:** Categorization for SC resilience to epidemic outbreaks

Category	Components
Systems	Structures, resources, capacities, interactions (responses, coordination)
Process	Distribution, transportation, procurement, production, resources allocation, flexibility
Control	Inventory control, sourcing control, manufacturing control, resilience as KPI in optimization models
Recovery	Manufacturing production, human labor, transportation network, suppliers, production flexibility

A set of critical components of SC resilience can be selected for each category. Besides, it should be noted that all the categories coming into play in the epidemic outbreak are interralated and correlated (e.g., preparedness, response, recovery). As such, faced with important disruptions caused by epidemics, SCs need to reinforce their resilience, including throught increased SC viability (Ivanov and Dolgui 2020b).

The available systems are considered a critical aspect of resilience performance by the literature (Aven 2017; Ivanov and Dolgui 2019). They encompass physical and digital components when it comes to SCs (Queiroz et al. 2019a), and support the various interactions of the epidemic outbreak.

The process category is focused on the interplay between flexibility and product management (Dolgui and Proth 2010; Ivanov and Dolgui 2019), as acknowledged by the relevant literature. This category calls for a detailed network for the flow of products while avoiding shortages. One efficient strategy for coping with these is related to the process redundancy (Ivanov and Dolgui 2019).

The control theory is a well-established conceptualization scheme that has been successfully used in the area of SC disruption and resilience (Ivanov et al. 2016, 2017; Spiegler et al. 2016). Severe epidemic outbreaks, including the COVID-19, fall in this category and are amplified, more than ever, by the complexities of the system’s dynamics. Consequently, new control policies to inventory, sourcing and manufacturing, coupled with the resilience of the KPI’s in optimization models, are required.

The recovery stage plays a fundamental role in SC resilience (Ivanov and Dolgui 2019; Elluru et al. 2019). It includes different policies for network reconfiguration (Ivanov et al. 2016), with the engagement and interaction of internal and external resources (e.g., manufacturing production, human labor, transportation network, suppliers, and production flexibility). It should be noted that because of shortages (human labor, products) and restrictions on the transportation network (World Economic Forum—WEF 2020a), recovering from disruptions caused by the pandemic vary according to regional policies and may face some delays. The recovery process could be accelerated by relying on alternative sources, along with the adoption of production flexibility strategies (World Economic Forum—WEF 2020a). For instance, by streamlining their production systems, manufacturing firms could produce more medical supplies to shorten the impacts of epidemics on SCs (World Economic Forum—WEF 2020a).

## Conclusion

The coronavirus (COVID-19) outbreak shows that pandemics and epidemics can seriously wreak havoc on supply chains (SC) around the globe. In this study, we presented a systematic analysis of the impacts of epidemic outbreaks on SCs guided by a structured literature review that collated a unique set of publications on epidemic outbreak impacts on SCs. Utilizing the outcomes of our analysis, we tease out a series of open research questions that would not be observed otherwise.

In terms of findings and contributions, this study showed that the interplay between SCs and epidemic outbreaks has so far traditionally focused on resource allocation problems and supply medicals distribution, using optimization approaches and epidemic models (Büyüktahtakın et al. 2018; Mamani et al. 2013; Rachaniotis et al. 2012). It has also revealed that such an interplay has been dominated by the influenza epidemic, though other relevant epidemic outbreaks are being reported in the SC context.

In this respect, the COVID-19 pandemic is already devastating global SCs. It should be recalled that investigating the impacts of epidemic outbreaks on SCs is a new but robust research stream (Ivanov 2020a). This study ended up providing an insightful and challenging research agenda to scholars and practitioners interested in exploring more deeply the effects of epidemic outbreaks on SCs. In this vein, we identified robust literature gaps and open question opportunities, which we classified in three clusters (modeling, technology, and organizational).

Most centrally, we proposed a framework of OSCM at the times of COVID-19 pandemic that spans six perspectives, i.e., adaptation, digitalization, preparedness, recovery, ripple effect, and sustainability. Despite the development of novel classifications and categorizations as well as articulation of novel theoretical tensions, our study harbors a number of limitations. The query used to search the keywords on the databases could be a barrier in exploring other SC-related themes. In the future, additional studies could adopt and extend our protocol for much better results. In addition, this work has been limited by the scarcity of research about the effects of epidemic outbreaks on SCs. Only 32 documents were found to fully meet our research protocol. Furthermore, as the COVID-19 effects on SCs are still going on and look increasingly devastating, there is not much room for comparative analysis. Considering such limitations, especially in the literature gap related to the effects of epidemic outbreaks on SCs, we have proposed research agenda for future studies.

In future, one promising research avenue is to frame the analyses of SC behaviors during and after the pandemic in the vein of *viability*. Viability nicely integrates resilience, adaptation, and sustainability views the importance of which in the analysis of SCs under pandemic conditions has been noted and reported (Ivanov 2020b; Ivanov and Dolgui 2020b; Sarkis et al. 2020). Another interesting research avenue is to enrich the OSCM methodical variety by the methods from other disciplines; e.g., to investigate the application of SEIR and epidemic diffusion models to the analysis of SC ripple effect or to study the applications of ecological modelling to SC viability. These and other directions open new, at times unforeseen research avenues where the OSCM can make substantial contributions to theory and practice in order to help firms to remain impactful and relevant during and after the COVID-19 pandemic.
